# Stellate ganglion block for ventricular arrhythmia disease states: Emerging treatment options

**DOI:** 10.1016/j.jatmed.2026.09.001

**Published:** 2026-10-10

**Authors:** Ahmed I. Anwar, Fatima Iqbal, Walid K. Mohammed, Alexander S. Harris, Emily A. Francis, Aishwarya Reddy, Henry Liu, Alan D. Kaye

**Affiliations:** a School of Medicine, Louisiana State University Health Sciences Center at Shreveport, Shreveport, LA 71103, USA; b Department of Cardiology, Ochsner Medical Center, New Orleans, LA 70121, USA; c Department of Anesthesiology Louisiana State University Health Sciences Center, Shreveport, LA 71103, USA; d Department of Anesthesiology & Critical Care Perelman School of Medicine, The University of Pennsylvania, 3400 Spruce Street, Philadelphia, PA 19104, USA

**Keywords:** Stellate, Ganglion, Arrhythmia, Autonomic, Nervous system, Electrical storm

## Abstract

Autonomic modulation has emerged as an effective therapeutic target in the management of ventricular arrhythmias and electrical storm (e.g., refractory ventricular arrhythmias), in a background of growing evidence of the link between the autonomic nervous system and arrhythmias. Stellate ganglion blockade (SGB) is a minimally invasive procedure targeting the stellate ganglion with an anesthetic agent that has emerged as a temporizing treatment option for management of refractory and hemodynamically unstable ventricular arrhythmias and electrical storm (ES). SGB which can be performed with fluoroscopy-guidance or at the bedside with anatomic landmark approach or ultrasound guidance, has shown potential efficacy in short-term reduction of arrhythmic burden and termination of electrical storm, based on data from case series and observational studies. In the current review, we highlight the role of the autonomic nervous system in arrhythmogenesis and the clinical applications as well as current limitations and future directions of SGB in management of ventricular arrhythmia and electrical storm.

## Introduction

Ventricular arrhythmias are associated with an increased risk of death, including sudden cardiac death (SCD), and therefore contribute significantly to morbidity and mortality.^1^ Traditional management of ventricular arrhythmias and electrical storm, defined as three or more episodes of sustained or treated ventricular tachyarrhythmias in a 24-h period, has constituted pharmacological management, mechanical circulatory support (MCS), deep anesthesia, catheter ablation as well as autonomic neuromodulation. The role of the autonomic nervous system in arrhythmogenesis has been well established, with beta-adrenergic receptor stimulation leading to arrhythmic triggers such as afterdepolarization and abnormal automaticity.^2^ In pathologic states of heart failure and myocardial infarction, autonomic dysregulation arising from sympathetic remodeling can lead to sympathetic overdrive, promoting arrhythmogenesis.^3^

The autonomic nervous system is therefore a therapeutic avenue for the management of arrhythmias, and has been targeted using various procedures, including thoracic epidural anesthesia, vagal nerve stimulation, renal sympathetic denervation as well as tragus nerve stimulation for acute neuromodulation for the control of stimulant use disorder and electrical storm.^4^ In recent years, stellate ganglion block (SGB) has emerged as a promising tool for the management of refractory and hemodynamically unstable ventricular arrhythmias, particularly when first-line options have either failed or are not suitable. The stellate ganglia, present bilaterally, are a collection of sympathetic ganglia which provide autonomic sympathetic innervation to the heart, including the conduction system and the contractile myocardium.^5^

SGB has historically been performed to treat several conditions including peripheral vascular disease, complex regional pain syndrome and posttraumatic stress disorder.^6^, ^7^ Overactivity of the stellate ganglion, resulting in increased levels of co-transmitters such as norepinephrine, has been linked to promotion of arrhythmogenesis.^8^, ^9^ By blocking the stellate ganglion with anesthetic agents, sympathetic tone to the heart is decreased, allowing for suppression of arrhythmogenesis. Currently, several case reports, case series and retrospective review studies have described the role of SGB in the management of ventricular arrhythmias.^7^, ^10^, ^11^, ^12^

Other modalities that have included manipulation of the stellate ganglion for the control of arrhythmias and electrical storm include surgical stellectomy as well as transcutaneous magnetic stimulation of the stellate ganglion.^13^ In this review we discuss the mechanisms, efficacy, safety and limitations of SGB for ventricular arrhythmias and electrical storm.

### Acute vs chronic neuromodulation framework in ventricular arrhythmias

The autonomic nervous system is recognized as a target for ventricular arrhythmias, particularly when antiarrhythmic medications, ablation, or other conventional therapies used for arrhythmias fail to provide control over the ventricular arrhythmia.^14^ These neuromodulator therapies can be valuable for patients with heart disease, in whom ventricular arrhythmia can recur despite therapy and ablation, both of which are common treatment options, and in whom some arrhythmogenic substrates remain difficult to target with current ablation technology.^14^ Acute neuromodulation aims to provide rapid suppression of sympathetic activity during periods of electrical instability, which include electrical storm or ventricular tachycardia that is refractory. The primary goal of this treatment is stabilizing patients by interrupting the ongoing arrhythmogenesis.^14^, ^15^

Additionally, thoracic epidural anesthesia (TEA) represents an acute neuromodulator strategy that reduces ventricular tachycardia burden in selected patients with refractory ventricular arrhythmias, with clinical benefit being observed after initiation.^15^ Similar to SGB, TEA is an acute strategy that can also serve as a bridge in patients who have refractory arrhythmias. Acute neuromodulation is a bridge strategy, which allows for recovery. In contrast, chronic neuromodulator interventions, such as cardiac sympathetic denervation (CSD), is intended to provide sustained modification of sympathetic input to the heart.^16^, ^17^ Evidence suggests that CSD provides long term arrhythmia control, especially in the bilateral setting.^16^, ^17^ SGB also offers a less invasive, but temporary, approach, while CSD has more durable sympathetic denervation as well as a longer-term arrhythmia control. The distinction between chronic and acute neuromodulation is important because the goals of therapy are different, with acute interventions focusing on the immediate suppression of the arrhythmia at hand, while chronic interventions are aimed at reducing long term recurrence.^15^, ^16^, ^17^ SGB is a temporary neuromodulator therapy, and emphasis should be placed on its short term outcomes including reduction in arrhythmia burden, and termination of electrical storm, rather than long term durability which is accomplished by interventions such as CSD.^15^, ^16^, ^17^ This distinction of acute versus chronic is seen in Table 1.

**Table 1:** Comparison between autonomic modulation strategies.

**Feature**	**SGB**	**TEA**	**CSD**
Timing	Acute	Acute	Chronic
Primary role	Bridge therapy	Bridge therapy	Prevention of recurrence
Invasiveness	Minimal	Neuraxial	Surgical
Duration	Short-term	Short-term	Long-term
Limitation	Temporary effect	Temporary effect	More invasive

### Stellate ganglion anatomy and physiologic function

The stellate ganglion is a star-shaped bilateral structure consisting of sympathetic nerves in the anterior, lower part of the neck in front of the first rib. In up to 80% of the population, it forms as a roughly 2.5 × 1 × 0.5 cm structure through the fusion of the inferior cervical sympathetic ganglion and the first thoracic sympathetic ganglion.^18^ Although this dimension may vary throughout the population, its location, anterolateral to the longus colli muscle, medial to the scalene muscles, anterior to the transverse process of the C7 cervical vertebra, and bounded medially by the trachea, esophagus, and vertebral column, tends to be consistent.

The cardiac sympathetic preganglionic neurons originate in the intermediate zone of the thoracic spinal cord and enter the sympathetic trunk to synapse on postganglionic neurons in multiple cervicothoracic ganglia, including the stellate ganglion.^3^ Postganglionic sympathetic neurons originating from the stellate ganglion merge with preganglionic parasympathetic nerves to form the cardiac nerves, which then form the cardiac plexus on the epicardium, innervating components of the conduction system, including the sinoatrial and atrioventricular node, as well as the contractile myocardium.

The right and left stellate ganglia provide innervation to different regions of the heart, with the right stellate ganglion innervating the sinoatrial node of the heart and the left stellate ganglion providing innervation to the ventricular myocardium.^19^ Additionally, the right stellate ganglion provides innervation to the anterior surface of the heart while the left stellate ganglion provides innervation to the posterior surface of the heart.^19^ As such, the right stellate ganglion has been shown to have effects on cardiac chronotropy while the left stellate ganglion has effects on inotropy. Prior research has also revealed that compared to the right stellate ganglion, the left stellate ganglion provides greater sympathetic tone to the myocardium.^20^ These territories are not strictly discrete, and the left and right innervation territories overlap.

Norepinephrine exerts these effects through activation of the cardiac β-AR receptors, subsequently leading to increased levels of sarcoplasmic reticulum Ca^2+^.^21^ In turn, increased Ca^2+^ leak during diastole leads to depolarizing currents and triggered activity including early afterdepolarizations (EADs) and delayed afterdepolarizations (DADs).^22^ Excess release of norepinephrine, in states of sympathetic hyperactivity, therefore, leads to triggered activity, increased automaticity as well as increased risk for reentrant circuits, all mechanisms that support ventricular arrhythmogenesis.^21^, ^23^

In addition to norepinephrine, sympathetic activation can lead to elevated levels of other co-transmitters, including neuropeptide y which can also lead to DADs.^24^ Factors that trigger sympathetic hyperactivity include physical and emotional stress, electrical shocks as well as cardiac pathologic states including heart failure and myocardial infarction.^25^ Additionally, inflammation has been postulated to further increase the risk of arrhythmias by increased activation of the stellate ganglion and release of neurotransmitters, highlighting the role of neuroimmunology in ventricular arrhythmogenesis.^26^, ^27^

Additionally, studies have identified recurrent laryngeal nerve block, phrenic nerve involvement, and vascular injury as adverse effects associated with SGB, necessitating continuous hemodynamic and respiratory monitoring, especially in critically ill patients.^28^ Additionally, in regards to safety and potential complications of SGB, studies have found that recurrent laryngeal nerve block, phrenic nerve injury, and vascular injury are potential adverse effects that may arise after having a SGB procedure.^29^ These side effects and complications that can arise with this procedure again demonstrate why continuous monitoring is important.^29^ There are other complications as well that have been reported to arise, and these include pneumothorax, intravascular injection, and injury to the structures that are adjacent, and one report found that approximately 1.7 per 1000 procedures had severe complications.^29^ The use of ECG and vital monitoring is important due to these potential complications.^6^ Additionally, ultrasound guidance may improve the safety of SGB by allowing for visualization of the surrounding structures.^30^

### Mechanism of SGB for ventricular arrhythmias and electrical storm

Percutaneous SGB has been described with the use of ultrasound guidance, fluoroscopy or landmark-driven without imaging assistance. While fluoroscopy is useful for the identification of bony structures, ultrasound allows for identification of the correct fascial plane for placement of needle tip and allows for identification of surrounding structures, therefore leading to decreased risk of injury to the esophagus, vertebral artery and the inferior thyroid vessels.^31^ Ultrasound-guided approach for SGB additionally allows for use of small volume of anesthetic agent through robust visualization of fascial planes.

Before SBG can be performed, assessment of vital signs and hemodynamic status is imperative. This assessment involves obtaining a complete history, medication list, electrocardiogram, blood tests, cardiac imaging, and documentation of ventricular arrhythmias.^4^ The process of performing a blockade of the stellate ganglion then begins with anesthetization.^4^ SGB is commonly performed at the C6 vertebra level for the anesthetic agent to caudally reach the stellate ganglion and avoid the vertebral artery.^32^ Advancement and placement of the local anesthetic at this level, instead of at the stellate ganglion directly, reduces complications and increases the chances of a successful block.^32^

Once the C6 vertebra has been visualized, the needle is guided by the ultrasound until its tip is between the longus colli muscle and the carotid artery.^20^ Doppler imaging can also be used for visualizing blood vessels to minimize the risk of puncture. Once the needle is placed in the right position, 8–10 mL of 0.2% ropivacaine is commonly injected.^20^ Ropivacaine is used more commonly than bupivacaine because of its longer duration of action (reported to range from 2 to 8 h), and a lower risk of cardiotoxicity.^20^ SGB can be performed either unilaterally or bilaterally. Prior studies have revealed that left SGB was as effective as bilateral SGB for control of ventricular arrhythmias.^33^, ^34^

A retrospective study by Sanghai et al. additionally revealed that while infusion of anesthetic agent with both single injection and continuous infusion was safe, continuous infusion of anesthetic agent resulted in a greater reduction of stimulant use disorder burden while reducing the need for repeat procedures.^35^ Following SGB, while blood levels of the local anesthetic decrease within h, suppression of ventricular arrhythmias lasts for days following the procedure.^20^

Acute neuromodulation strategies are intended to provide rapid suppression of sympathetic ventricular arrhythmias during an electrical storm and an example of an acute strategy is the SGBs, which are performed as either a single injection or a continuous catheter based infusion.^36^, ^37^ Continuous SGB is considered a safe and capable strategy for sustained arrhythmia suppression particularly for prolonged modulation.^36^ Additionally, neuromodulator therapies should be integrated into a multidisciplinary treatment strategy for refractory ventricular arrhythmia. In these patients, acute autonomic interventions serve as an important component of comprehensive care.^37^

Following SGB, measuring success is a priority. Although a successful block is the goal, there are few reliable markers that measure high sensitivity and specificity. Some markers that have been studied include a rise in temperature in the ipsilateral hand, the presence of Horner’s syndrome if above T1, and an increase in the perfusion index.^20^

### Limitations of stellate ganglion block for ventricular arrhythmias and electrical storm

Although SGB has emerged as an important neuromodulator strategy for the management of refractory ventricular arrhythmias and electrical storm, particularly in the acute setting, several important limitations and potential harms restrict its current clinical utility.^38^ One major limitation of SGB is uncertainty regarding the durability and completeness of sympathetic denervation achieved with percutaneous techniques.

Negative effects associated with SGB are primarily related to incomplete or transient sympathetic interruption rather than direct procedural complications. Horner’s syndrome is commonly used as an acute procedural endpoint, yet this finding only confirms blockade of ocular sympathetic fibers and does not reliably indicate effective cardiac sympathetic denervation. Importantly, the resolution of Horner’s syndrome in reported patients suggests that SG injury may be temporary, raising concern that arrhythmia suppression may diminish over time.^38^ In addition, the absence of functional testing, such as assessment of skin temperature or sweating patterns, limits the ability to confirm whether downstream thoracic sympathetic pathways have been adequately impacted.

Evidence supporting SGB is also limited by small study populations and confounding variables. Additionally, some patients underwent ventricular tachycardia ablation or recent coronary revascularization, making it difficult to isolate the independent effect of SGB of arrhythmia outcomes.

Overall, while SGB represents a potentially useful therapy for selected high-risk patients, limitations, including uncertain permanence, incomplete sympathetic coverage, and limited supporting evidence, currently restrict its role.

### Studies evaluating outcomes of stellate ganglion blocks in ventricular arrhythmias

Across observational studies and meta-analyses, temporary sympathetic modulation with SGB has consistently demonstrated rapid and clinically meaningful reductions in ventricular arrhythmia burden among patients with refractory ventricular tachycardia, ventricular fibrillation, and electrical storm.

The largest and most robust real-world evidence comes from contemporary multicenter cohorts. In the STAR study, Savastano et al. reported that percutaneous SGB achieved a ≥ 50% reduction in arrhythmic events within 12 h in 92% of patients, with a reduction in median arrhythmia episodes from six to zero, when comparing the 12 h before the first SGB with the 12 h after the last procedure, supporting SGB as a highly effective acute rescue therapy in electrical storm.^39^

Similarly, Chouairi et al. demonstrated significant reductions in both ventricular tachycardia/ventricular fibrillation episodes and defibrillation requirements within 24 h following bedside SGB across 117 patients, reinforcing the reproducibility and feasibility of this intervention in critically ill populations.^11^ Smaller single-center and retrospective studies further corroborate these findings, showing that unilateral or bilateral ultrasound-guided SGB can suppress arrhythmias and markedly reduce implantable cardioverter-defibrillator (ICD) shocks for 24–96 h post-procedure.^33^, ^40^, ^41^ Technique-specific variations appear to influence durability of effect, as continuous infusion strategies using catheter-based SGB have been associated with more sustained arrhythmia control compared with single-injection approaches.^35^, ^42^

Novel sympathetic blockade approaches, such as ultrasound-guided proximal intercostal block, have also shown promising efficacy, with substantial ventricular tachycardia/ventricular fibrillation suppression and acceptable safety profiles, suggesting potential alternatives when traditional SGB is not feasible.^43^ A recent meta-analysis encompassing over 500 patients confirmed a consistent reduction in ventricular arrhythmia burden and complete electrical storm resolution in approximately two-thirds of treated patients, although short-term mortality (30-day) remained high (64.6%), reflecting the advanced structural heart disease and critical illness of this population rather than procedural inefficacy.^12^

Collectively, these data support SGB as an effective temporizing therapy for refractory ventricular arrhythmias, capable of rapidly stabilizing patients and reducing defibrillation burden, while highlighting the need for randomized trials and standardized protocols to better define its role, optimal technique, and long-term impact within comprehensive arrhythmia management strategies (Table 2).^11^, ^12^, ^35^, ^39^

**Table 2:** Key studies evaluating stellate ganglion block for ventricular arrhythmias.

**Study**	**Design, n**	**Technique**	**Key Outcome**
Savastano et al.^39^	Multicenter, n = 131	Percutaneous SGB	≥ 50% reduction in arrhythmic events
Chouairi et al.^11^	Multicenter, n = 117	Bedside SGB	Reduced ventricular tachycardia / ventricular fibrillation episodes
Sanghai et al.^35^	Retrospective, n = 18	Left sided SGB, single injection, continuous infusion	Ventricular arrhythmia burden reduced 45% with single injection and 94% with continuous infusion.
Motazedian et al.^12^	n > 500 patients	Various SGB techniques	Electrical storm resolution in 2/3 of patients

### Stellate ganglion block in cardiac arrest and peri-arrest ventricular arrhythmias

SGB has been seen as a therapy for refractory ventricular dysrhythmias, especially when a conventional resuscitation measure failed to stop the tachyarrhythmia.^44^ Refractory ventricular dysrhythmia, also known as an electrical storm, is characterized by three or more episodes of ventricular arrhythmia within a 24-h period, and these dysrhythmias are resistant to the standard treatment. In a two patient emergency department case series, ultrasound-guided SGB performed during a cardiac arrest was associated with successful termination of the refractory ventricular tachydysrhythmia after the standard resuscitative intervention failed.^44^ This case suggests that physicians may consider the SGB as an adjunctive intervention for patients with resistant tachyarrhythmia during cardiac arrest. Another case report described cardiac arrest due to ventricular fibrillation that demonstrated a successful return to normal circulation after a combination of resuscitative balloon occlusion and intra-arrest left SGB, as well as neurological recovery after this intervention.^45^

SGB has also been used to reduce recurrence of ventricular arrhythmia in multiple settings, and in a patient with myocarditis and refractory ventricular fibrillation, SGB facilitated restoration of a stable sinus rhythm after unsuccessful defibrillation.^46^ The rationale for using autonomic modulation for refractory ventricular arrhythmia is supported by evidence that adrenergic stimulation that is more than normal, or excessive, can contribute to arrhythmogenesis, which is also seen in studies evaluating beta blockade during ventricular fibrillation.^47^

### Future directions

Current data regarding the effectiveness of SGB for management of ventricular arrhythmias and electrical storm is based on case reports, case series and observational studies. To date, no randomized clinical trials have been carried out to compare the effectiveness of SGB to traditional methods of management (Table 2).

At present, the GANGlion Stellate Block for Treatment of Electric stoRm Trial (GANSTER trial) is an ongoing double-blinded randomized control trial that aims to evaluate the efficacy of left SGB with Bupivacaine compared to placebo in patients with drug-refractory electrical storm (NCT05078684).^48^ Other areas of exploration include comparison of SGB with radiofrequency ablation (RFA) and cryoablation of stellate ganglion, comparison of efficacy of bilateral versus unilateral blockade, further investigation into identification of patients most likely to benefit from SGB for stimulant use disorder and electrical storm.

Additionally, there is currently a lack of standardized patient selection criteria and procedural protocols for SGB in the management of stimulant use disorder and electrical storm. Indeed, anisocoria, which has classically been used as a marker of effectiveness, has proven to be unreliable, highlighting the need for reliable markers indicative of effectiveness. Similarly, while both single injection and continuous infusion of anesthetic agents for SGB have demonstrated efficacy, there is a lack of robust data comparing the two methods. It is also important to identify the responders to SGB, the biomarkers that would allow for better patient selection, and the role of combination strategies. This new information would allow for better patient selection and better safety for the patients who are selected and thought to respond well to this treatment (Table 3).

**Table 3:** Advantages, limitations, and future directions of SGB.

**Advantages**	**Limitations**	**Future directions**
Bedside, minimally invasive	Lack of randomized trials	GANSTER trial results
Bridge therapy	No reliable marker for denervation	Standardized protocols
Safe and well tolerated	Small observational studies	Long term recurrence outcomes

## Conclusions

The role of the autonomic nervous system in genesis of ventricular arrhythmias is well-established. In recent years, neuromodulation has emerged as an effective avenue in the management of refractory ventricular arrhythmias and electrical storm. As the stellate ganglion provides sympathetic innervation to the heart, SGB, which can be performed at the bedside with ultrasound-guidance or with anatomical landmark guidance, is a viable neuromodulator treatment option for hemodynamically unstable and life-threatening ventricular arrhythmias and electrical storm, which are associated with a high burden of morbidity and mortality. It represents a minimally invasive procedure that can be carried out at the bedside, particularly for hemodynamically unstable patients and has an overall safe and tolerable side effect profile.

Evidence for its clinical utility is based on data from case reports, case series and observational studies, albeit with a lack of randomized clinical trials evaluating its efficacy. Current evidence also suggests that SGB may provide meaningful short-term reductions in arrhythmic burden for patients and it primarily serves as a bridge therapy for patients with refractory ventricular arrhythmias and electrical storm. The current data suggests that SGB is highly effective with significant short-term reduction of arrhythmic burden. However, data regarding its long-term efficacy, including effect on morbidity and mortality, remains uncertain. Several other areas remain poorly defined as well, including patient populations most likely to benefit from SGB and standardized methodology and protocols. Overall, percutaneous SGB is a promising treatment modality in the management of ventricular arrhythmias and electrical storm. Further research, including data from randomized clinical trials, demonstrating its long-term efficacy, safety and effect on morbidity and mortality would undoubtedly cement its role further in the management of ventricular arrhythmia and electrical storm.

## CRediT authorship contribution statement

**Alan D. Kaye:** Writing – review & editing, Writing – original draft, Visualization, Validation, Supervision, Software, Resources, Project administration, Methodology, Investigation, Formal analysis, Data curation, Conceptualization. **Henry Liu:** Writing – review & editing, Supervision. **Aishwarya Reddy:** Writing – review & editing, Writing – original draft, Visualization, Validation, Supervision, Software, Resources, Project administration, Methodology, Investigation, Formal analysis, Data curation, Conceptualization. **Emily A. Francis:** Writing – review & editing, Writing – original draft, Visualization, Validation, Supervision, Software, Resources, Project administration, Methodology, Investigation, Formal analysis, Data curation, Conceptualization. **Alexander S. Harris:** Writing – review & editing, Writing – original draft, Visualization, Validation, Supervision, Software, Resources, Project administration, Methodology, Investigation, Formal analysis, Data curation, Conceptualization. **Walid K. Mohammed:** Writing – review & editing, Writing – original draft, Visualization, Validation, Supervision, Software, Resources, Project administration, Methodology, Investigation, Formal analysis, Data curation, Conceptualization. **Fatima Iqbal:** Writing – review & editing, Writing – original draft, Visualization, Validation, Supervision, Software, Resources, Project administration, Methodology, Investigation, Formal analysis, Data curation, Conceptualization. **Ahmed I. Anwar:** Writing – review & editing, Writing – original draft, Visualization, Validation, Supervision, Software, Resources, Project administration, Methodology, Investigation, Formal analysis, Data curation, Conceptualization. All authors have read and agreed to the published version of the manuscript.

## Consent for publication

Not applicable.

## Ethical statement

Not applicable.

## Funding

This research received no external funding.

## Declaration of generative AI and AI-assisted technologies in the writing process

The authors used Open AI for grammar clarity. The authors reviewed and edited the output as needed and take full responsibility for the content of the published article.

## Declaration of competing interest

Henry Liu (Editor-in-Chief) and Alan D. Kaye (Associate Editor) of Journal of Anesthesia and Translational Medicine were not involved in the editorial review or the decision to publish this article.

## Data Availability

All data cited in this review are publicly available through the original publications listed in the references. Source data for figures are provided with this paper.
